# Short-Term Effects of Kinesio Taping in the Treatment of Latent and Active Upper Trapezius Trigger Points: two Prospective, Randomized, Sham-Controlled Trials

**DOI:** 10.1038/s41598-019-51146-4

**Published:** 2019-10-09

**Authors:** Yolanda Noguera-Iturbe, Javier Martínez-Gramage, Francisco Javier Montañez-Aguilera, José Casaña, Juan Francisco Lisón

**Affiliations:** 1 Department of Physiotherapy, Faculty of Health Sciences, Universidad Cardenal Herrera-CEU, CEU Universities, Valencia, Spain; 2 Exercise Intervention for Health Research Group (EXINH-RG), Department of Physiotherapy, University of Valencia, Valencia, Spain; 3 Department of Medicine, Faculty of Health Sciences, Universidad Cardenal Herrera-CEU, CEU Universities, Valencia, Spain; 4 CIBER of Physiopathology of Obesity and Nutrition CIBERobn, CB06/03 Instituto de Salud Carlos III, Valencia, Spain

**Keywords:** Pain management, Rehabilitation

## Abstract

The presence of myofascial trigger points (MTrPs) is one of the most common causes of musculoskeletal problems and may lead to limited professional activity. Among the various treatment methods proposed for MTrPs, Kinesio Taping (KT) is a non-invasive, painless, and less time-consuming method with fewer side effects that has become widely used as a therapeutic tool in a variety of prevention and rehabilitation protocols. The aim of the study was to evaluate the immediate and short-term efficacy of the space correction KT technique in patients with latent or active MTrPs in the upper trapezius muscle. Two parallel randomized sham-controlled trials were simultaneously executed: in trial A, ninety-seven participants with latent MTrPs were randomly assigned to either the KT (n = 51) or sham (n = 46) group; in trial B, thirty-seven participants with active MTrPs were assigned to the KT (n = 20) or sham (n = 17) group. The primary outcome was pressure pain threshold (PPT) in the upper trapezius muscle, measured with algometry. Secondary outcomes included the active range of motion (ROM) of the cervical spine (lateral flexion and rotation), measured with a cervical ROM goniometer. In each trial, two-way ANOVA tests were used to compare the study effects on the outcome measures between the groups, with time serving as the intra-group factor (baseline, immediately, and 72 h after the application) and the intervention type (KT and sham) as the between-group factor. At 72 h, participants receiving KT did not show significant differences in PPT (trial A: mean difference −1.8 N; 95% CI: [−8.1, 4.4], trial B: mean difference −1.2 N; 95% CI: [−7.4, 5.1]), cervical lateral flexion (trial A: mean difference 0.2 degrees; 95% CI: [−2.7, 3.1], trial B: mean difference −2.4 degrees; 95% CI: [−8.4, 3.6]), and cervical rotation (trial A: mean difference 3.7 degrees; 95% CI: [−0.1, 7.5], trial B: mean difference 1.4 degrees; 95% CI: [−5.7, 8.4]), compared to the sham groups. Thus, the results of this study do not support the use of the space correction KT technique to treat patients with latent or active myofascial trigger points in the upper trapezius muscle.

## Introduction

Musculoskeletal problems are often caused by myofascial pain syndrome^1^ and frequently cause morbidity — affecting up to 85% of adults at least once in their lifetime^2^. This syndrome results in the presentation of myofascial trigger points (MTrPs), i.e. hyperirritable spots found in tense skeletal muscle bands^3^ that may lead to muscle dysfunction, thus limiting work and leisure activities^4–6^. Depending on their clinical characteristics, MTrPs may be classified as active or latent^7^: the latter refers to points at which pain is triggered upon direct pressure application while persistent pain is caused by active trigger points even when manual pressure is not applied^8^. Although latent trigger points are not the cause of continued pain, they restrict movement, induce early fatigue and muscle weakness^9–13^, and can progress to become active trigger points^14^.

MTrPs are most commonly found in the upper trapezius (UT) muscle^15^. Interestingly, the UT also has the lowest pressure-pain threshold (PPT) and is the most sensitive to MTrPs^16^, perhaps because it is constantly working against gravity to maintain an erect head and neck position^17^. Indeed, MTrPs within the UT may cause neck pain and stiffness, restricted cervical spine range of motion (ROM), and headaches^5,8,18^. Specifically, latent MTrPs in the UT are able to disturb muscle movement patterns and can be the root of pathologies including cramping, weakness, and decreased muscle strength^19^. Therefore, latent and active MTrPs in the UT must be addressed and adequately treated to prevent additional complications.

Several therapies have been proposed for MTrPs, including Kinesio Tape (KT), a relatively new method that has become widely used as a therapeutic tool in a variety of prevention and rehabilitation protocols^20–26^, and even very recently, in animals^27^. Moreover, it is non-invasive, painless, and less time-consuming than other options with fewer side effects. KT is an elastic-cotton adhesive tape which is latex-free and can be used on any joint or muscle^28^. It differs from other rigid tapes because it can be significantly stretched (by 130–140% of its original length), which reduces mechanical movement limitations and mimics skin in terms of its thickness and elasticity.

The exact mechanism by which KT functions remain unknown, though it is thought that its effectiveness may be mediated by cutaneous mechanoreceptors which would provide sensorimotor and proprioceptive feedback, and/or by mechanical restraint and inhibitory and excitatory nociceptive stimuli^26,29,30^. Various groups have shown that the use of KT can be beneficial, for example, by decreasing pain and muscular spasms^31–33^ or by increasing ROM^25,34^. However, very little research has been done to demonstrate the effect of KT in patients with cervical-region MTrPs^5,29,35–38^, and there is contradictory evidence about its effectiveness for neck conditions. Thus, in this study we performed two parallel randomized sham-controlled trials, to compare the short-term efficacy of KT and sham KT methods on UT muscle PPT and cervical ROM in patients with latent (trial A) and active (trial B) MTrPs.

## Methods

These prospective randomized, double-blind, sham-controlled trials (trial A, NCT02913963, 26/09/2016; and trial B, NCT02913976, 26/09/2016) were approved by the University Cardenal Herrera-CEU Human Ethics Committee and followed the ethical guidelines set out in the Declaration of Helsinki. The participant inclusion criteria were the presence of latent (trial A) or active (trial B) MTrPs in the UT muscle and an age between 18 and 65 years. The exclusion criteria were as follows: (1) prior neck or shoulder surgery; (2) diagnosis of fibromyalgia syndrome; (3) radiculopathy; (4) medical diagnosis of cervical osteoarthritis; (5) neck pain resulting from cervical whiplash or direct neck area trauma; (6) diagnosis of psychiatric disorders such as anxiety or depression; (7) pregnancy; (8) recent trigger-point injection or participation in a physical treatment program in the month prior; and (9) having already received treatment with KT.

A total of 97 volunteers with latent MTrPs were recruited at the University (trial A), and 37 with active MTrPs were recruited at different clinics in Elche (Spain; trial B). All participants read an information leaflet and then signed their informed consent to participation before starting the study.

**Figure 1:**
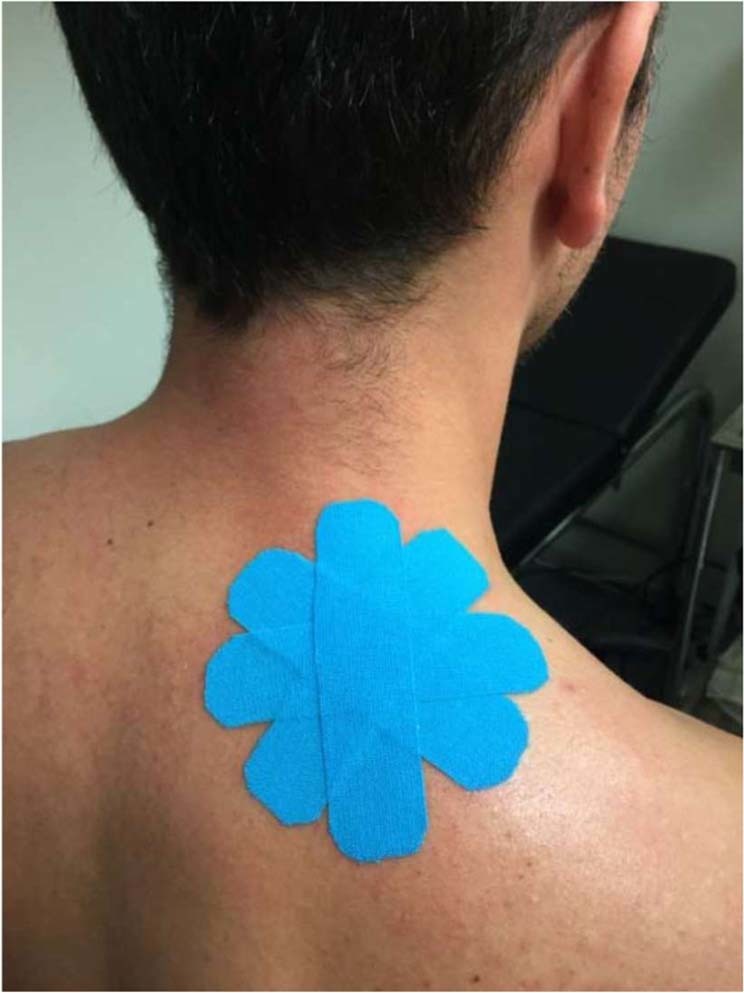
KT application (space-correction technique) on MTrPs in the upper trapezius.

The participants allocated to the KT intervention groups in trial A (latent MTrP) and in trial B (active MTrP) received the tape application once and the tape remained in place for 72 h, following a previously described standardized protocol^39^. The patients were seated erect, on a backless stool, with their back to the evaluator, their arms resting on their lap, their hips and knees bent at 90°, and their feet resting on the floor. Before KT application, their skin was shaved, cleaned with alcohol, and dried. In addition, an adhesive spray (Tuff Skin^©^ adherent tape) was applied to improve the permanency of the strips. KTs (Kinesio Tex Gold^©^) were applied to the UT muscles using four 10 cm “I” strips arranged in a star shape (space correction) directly above the MTrPs (Fig. 1). Each “I” strip was divided into three parts: two ends (1.5 cm each) without tension, and the middle (7 cm) with 25% of its available tension. Participants allocated to the sham groups received the same application (four “I” strips in a star shape) but applied with no tension. All applications were performed by the same researcher (certified KT3 physiotherapist with more than 10 years of clinical experience in treating MTrPs using KT). All participants (trials A and B) were evaluated before KT application, 15 min immediately after the application, and 72 h after the application.

The primary outcome was the PPT on the MTrP of the UT muscle, which was recorded with a pressure algometer (Commander TH, JTECH, Medical Industries^©^). The algometer was placed vertically at a right angle on the UT (dominant side) MTrP with the participant in a seated position. Pressure was applied at an increasing rate of 1 kg/second and participants were asked to indicate when the sensation of pressure had become painful. At this moment, the threshold (Newtons) was recorded from the algometer screen. This procedure was repeated three times for each participant with an interval of 30 s, and the mean value was used as the participant’s PPT for statistical analysis. The PPT is a valid and highly reliable tool in clinical studies of regarding myofascial and musculoskeletal pain^40,41^. Specifically, pressure algometry as an index for MTrP sensitivity has shown highly significant inter-rater and intra-rater experimenter reliability^42^.

Secondary outcomes included the active ROM of the cervical spine (lateral flexion and rotation), which was measured with the CROM goniometer (Performance Attainment Associates, Roseville, MN). The goniometer was placed on top of the head with the participant sitting on a chair, with both feet flat on the floor, hips and knees at 90° flexion, and buttocks positioned against the back of the chair. Participants were asked to move their heads as far as possible through the following movements: contralateral tilt relative to the studied trapezius muscle, and ipsilateral rotation tilt regarding the studied trapezius muscle. Each movement was performed three times, and averages of the resulting values were used to carry out the analyses. The CROM goniometer has been shown to exhibit excellent intratester reliability^43^.

In trial A, a pilot study was conducted with 20 participants, assigned randomly and equally to two groups (KT and sham tape). In order to determine the sample size, a power analysis was performed using the G*Power (v3.1.9.2) program; 40 patients per group would provide 90% statistical power at a 5% significance level (effect size *f* = 0.165) according to algometer scores between groups. To accommodate the expected dropout rate before the study’s completion, a total of 48 participants were included in each group.

Before the start of the trials, Researcher 1, who was not involved in either the recruitment or treatment of participants, organized the preparation of numbered, opaque, sealed envelopes containing the group allocation (for both trials independently, A and B). Researcher 2 generated the random sequences for both trials (based on simple randomization) using a computerized random number generator; this was concealed from all study personnel throughout the duration of the trials^44^. Upon enrollment in trial A, 97 participants with latent MTrPs were randomly assigned either to the KT (*n* = 51) or sham (*n* = 46) group. Similarly, in trial B, 37 participants with active MTrPs were randomly assigned to the KT (*n* = 20) or sham (*n* = 17) group. All outcome measures were recorded before, 15 min and 72 h after the application of KT by two trained physiotherapists who were blinded to the group allocation.

We analyzed our data using an intention-to-treat approach. In each trial, two-way mixed analysis of variance (ANOVA) tests were used to compare the study effects on the outcome measures between the groups, with time serving as the within-group factor (baseline and immediately after and 72 h after the application) and the intervention type (KT and sham) as the between-group factor. To analyze the effectiveness of the participants’ and evaluators’ blinding, we performed chi-squared tests. The statistical analysis was performed using SPSS Statistics version 19.0 (SPSS Inc., Chicago, IL^©^). A probability of *p* < 0.05 was considered statistically significant.

## Results

**Figure 2:**
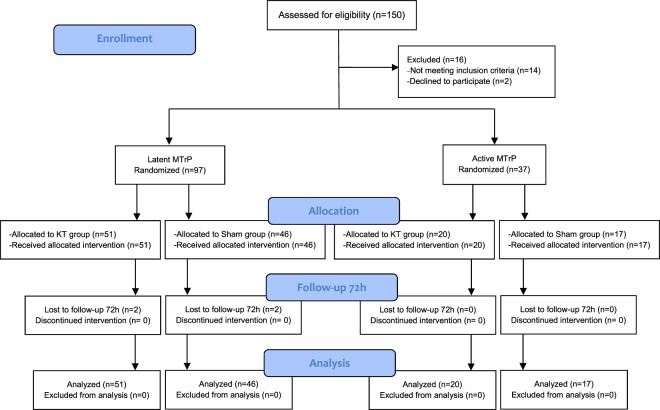
Flow of participants through the trial.

A total of 150 participants were recruited for this study; 16 were not allocated for randomization because they declined to participate (2) or did not meet the inclusion criteria: no MTrPs (9), musculoskeletal disorders of the neck (2), fibromyalgia (1), or prior treatment with KT (2); 134 participants with latent (97 in trial A) or active (37 in trial B) MTrPs were independently randomized. Figure 2 shows the progression of the participants through the trials and Table 1 shows their baseline characteristics.

**Table 1:** Baseline participant characteristics.

VARIABLES	Latent MTrP (trial A)		Active MTrP (trial B)	
	KT (*n* = 51)	ST (*n* = 46)	KT (*n* = 20)	ST (*n* = 17)
Age (years)	23.9 ± 7.8	26.2 ± 9.5	22.9 ± 7.1	21.7 ± 6.2
BMI (Kg/m^2^)	22.5 ± 3.0	24.2 ± 4.5	22.6 ± 4.5	22.8 ± 4.1
Men/Women	21/30	18/28	6/14	5/12
PPT (N)	32.9 ± 12.0	32.2 ± 12.1	25.6 ± 9.9	28.8 ± 9.3
CLF (degrees)	41.9 ± 7.9	39.7 ± 6.5	43.2 ± 7.3	45.3 ± 9.9
CR (degrees)	69.0 ± 10.0	65.6 ± 9.7	68.3 ± 14.0	71.2 ± 12.9

KT, Kinesio tape; ST, sham tape; MTrP, myofascial trigger point; BMI, body mass index; PPT, pressure pain threshold; CLF, cervical lateral flexion; CR, cervical rotation.

Data shown as the mean ± *SD* scores.

No differences in terms of age, BMI, sex, PPT, cervical lateral flexion, or cervical rotation were observed among groups (KT vs. sham) at baseline in either of the two trials and none of the participants reported any adverse events or intolerance to the intervention applied. The chi-squared test showed no differences between the groups, indicating that good participant and evaluator blinding was achieved.

The results of the two-way ANOVA did not show a significant time*intervention interaction effect on any outcome in either of the two trials (*p* > 0.05). In the latent MTrPs participants (trial A), the intragroup analysis showed a significant increase in the posttreatment PPT in both KT (4.1, 95% CI [1.3, 6.9] and sham 4.1, 95% CI [1.1, 7.0] groups. However, this significant intragroup effect disappeared at 72 h. No intragroup differences were found in the active ROM variables. Regarding the active MTrP participants (trial B), no intragroup differences were found in the PPT or active ROM (Table 2). The between-group analysis showed no significant differences for any comparison in either of the two trials (Table 3).

**Table 2:** Intragroup comparisons.

VARIABLES	Latent MTrP (trial A)							
	KT (*n* = 51)				ST (*n* = 46)			
	Posttreatment minus Baseline		Follow-up 72 h minus Baseline		Posttreatment minus Baseline		Follow-up 72 h minus Baseline	
	Diff. (95% CI)	*p*	Diff. (95% CI)	*p*	Diff. (95% CI)	*p*	Diff. (95% CI)	*p*
PPT (N)	4.1 (1.3 to 6.9)	0.002	1.7 (−2.3 to 5.6)	0.926	4.1 (1.1 to 7.0)	0.004	4.1 (0.0 to 8.3)	0.053
CLF (degrees)	1.4 (−0.3 to 3.0)	0.130	−0.3 (−2.2 to 1.6)	1	1.5 (−0.2 to 3.2)	0.108	1.7 (−0.3 to 3.7)	0.123
CR (degrees)	−0.1 (−2.1 to 2.0)	1	2.1 (−0.1 to 4.4)	0.071	1.6 (−0.6 to 3.8)	0.243	1.8 (−0.6 to 4.3)	0.194
**VARIABLES**	**Active MTrP (trial B)**							
	**KT (*n*** = **20)**				**ST (*n*** = **17)**			
	**Posttreatment minus Baseline**		**Follow-up 72 h minus Baseline**		**Posttreatment minus Baseline**		**Follow-up 72 h minus Baseline**	
	**Diff**. **(95% CI)**	***p***	**Diff**. **(95% CI)**	***p***	**Diff**. **(95% CI)**	***p***	**Diff**. **(95% CI)**	***p***
PPT (N)	1.9 (−2.5 to 6.2)	0.848	1 (−4.3 to 6.3)	1	1.8 (−2.9 to 6.5)	1	−1.0 (−6.7 to 4.7)	1
CLF (degrees)	−0.4 (−3.0 to 2.2)	1	−1.5 (−5.4 to 2.3)	0.946	0.7 (−2.1 to 3.5)	1	−1.2 (−5.3 to 3.0)	1
CR (degrees)	0.4 (−3.4 to 4.2)	1	3.2 (−0.7 to 7.2)	0.142	−1.8 (−6.0 to 2.5)	0.908	−1.0 (−5.4 to 3.4)	1

KT, Kinesio tape; ST, sham tape; MTrP, myofascial trigger point; PPT, pressure pain threshold; CLF, cervical lateral flexion; CR, cervical rotation; CI, confidence interval.

Data shown as the intragroup mean differences (95% CI).

**Table 3:** Between-group comparisons at posttreatment and at follow-up 72 h.

VARIABLES	Latent MTrP (trial A)				KT MINUS ST			
	Posttreatment		Follow-up 72 h		Posttreatment		Follow-up 72 h	
	KT	ST	KT	ST	Diff. (95% CI)	*P*	Diff. (95% CI)	*p*
PPT (N)	36.9 ± 13.0	36.3 ± 16.5	34.5 ± 13.3	36.4 ± 17.3	0.7 (−5.4 to 6.7)	0.830	−1.8 (−8.1 to 4.4)	0.561
CLF (degrees)	43.3 ± 8.5	41.2 ± 7.6	41.7 ± 6.9	41.5 ± 7.3	2.1 (−1.2 to 5.4)	0.216	0.2 (−2.7 to 3.1)	0.877
CR (degrees)	68.9 ± 9.7	67.2 ± 9.4	71.2 ± 10.1	67.5 ± 8.2	1.7 (−2.2 to 5.6)	0.390	3.7 (−0.1 to 7.5)	0.059
**VARIABLES**	**Active MTrP (trial B)**				**KT MINUS ST**			
	**Posttreatment**		**Follow-up 72 h**		**Posttreatment**		**Follow-up 72 h**	
	**KT**	**ST**	**KT**	**ST**	**Diff**. **(95% CI)**	***p***	**Diff**. **(95% CI)**	***p***
PPT (N)	27.5 ± 11.5	30.6 ± 10.8	26.7 ± 9.0	27.9 ± 9.7	−3.1 (−10.6 to 4.4)	0.408	−1.2 (−7.4 to 5.1)	0.702
CLF (degrees)	42.8 ± 7.4	46.0 ± 10.2	41.7 ± 6.9	44.1 ± 10.9	−3.1 (−9 to 2.7)	0.285	−2.4 (−8.4 to 3.6)	0.415
CR (degrees)	68.7 ± 14.5	69.4 ± 11.9	71.5 ± 9.7	70.2 ± 11.2	−0.7 (−9.9 to 8.4)	0.872	1.4 (−5.7 to 8.4)	0.701

KT, Kinesio tape; ST, sham tape; MTrP, myofascial trigger point; PPT, pressure pain threshold; CLF, cervical lateral flexion; CR, cervical rotation; CI, confidence interval.

Data are mean ± SD scores or between-group mean differences (95% CI). *p*-values were obtained from 2-way mixed analysis of variance (post hoc tests).

## Discussion

This study assessed the short-term effects of KT on PPT and cervical ROM in patients with latent (trial A) or active (trial B) UT MTrPs. Both randomized sham-controlled trials failed to identify any significant effects of KT on any outcome at 72 h, and only the latent MTrP trial participants showed a significant increase in PPT immediately after treatment in both the KT and sham groups — an effect which disappeared at 72 h.

In the literature, only a few studies have examined the effectiveness of KT in patients with cervical region MTrPs^5,29,35–38^. Because of the differences in the interventions applied (target muscle, taping method, concomitant treatments, etc.) and/or methodological differences in the design (different inclusion criteria [latent vs. active MTrPs], study variables, statistical design, sample size, etc.), these studies have reported different and sometimes contradictory results. One of the main factors that could influence the effects of KT is the technique (taping method) used which, according to a recent review^22^, may be related to the fact that faciliatory or inhibitory effects would be elicited depending on the taping direction. The amount of tension applied and the time the tape is left *in situ* may also influence the pain-reduction effect size. In our study, we applied KT on the UT muscle for 3 days by placing four “I” strips in a star shape on the MTrP itself to increase the space directly on the specific area of pain. The strips were placed with a tension of 25% in the experimental group and with no tension in the sham group. As far as we know, a similar technique has only been used in three other studies, as described below^5,35,36^.

Halski *et al*.^5^ applied KT to latent MTrPs of the UT using the space correction technique but with higher (50%) tape tension in a group of 25 volunteers over 3 days, compared to both a sham application (identical technique but with no strip tension, *n* = 24) and another taping modality (cross taping, *n* = 24). In contrast with our results, these authors reported a significant reduction in visual analogue scale (VAS) pain scores in all three groups, both immediately after the intervention (at 72 h) and 24 h after the treatment. These patients also showed a significant increase in their range of left lateral cervical flexion, although the authors did not clarify on which side of the UT muscle the different MTrPs had been identified. Furthermore, they concluded that KT application in all three groups did not influence resting UT muscle bioelectrical activity, and so might not produce reduced muscle tone in MTrP cases. The difference in tension of the strips in the two studies (50% vs 25% in ours), as well as the different instruments used to assess the effects of KT on pain (VAS vs PPT) and on cervical ROM (tape measurement vs algometry), could explain the differences between our results. In addition, taking into account the inclusion criteria that they used in their study (“being asymptomatic”, “latent MTrPs in the UT”), the high values of the VAS scores at baseline (7.2, 6.8 and 6.4 in cross taping, KT, and sham groups, respectively, on a scale of 10 cm) do bring up some questions. In contrast, Boonstra *et al*. found that in patients with chronic musculoskeletal pain, VAS scores of 3.4 or less corresponded to mild pain, scores of 3.5–7.4 represented moderate musculoskeletal pain, and those exceeding 7.5 were indicative of severe pain^45^. Finally, the statistical analysis by Halski *et al*.^5^ was limited to one-way ANOVA analysis, without studying time–group interactions.

Another study evaluated the short and long-term effects of KT application on MTrP PPTs in the gastrocnemius and UT muscles in a sample of 30 participants (15 each in the KT and sham groups)^35^. Contrary to our results, these authors stated that placing KTs directly on MTrPs could block the increased sensitivity usually felt straight after their application and could mitigate continued sensitization for up to 24 h. Although they applied the same KT method (space correction with “approximately 30% of the available tension”), unlike our study, the control group received the KT application close to, but not directly on the MTrPs. In addition, these authors did not explain whether these were latent or active MTrPs, and indeed, recognized that their study was limited both by their inexperience in evaluating MTrPs and by not implementing investigator group-allocation blinding.

Mohamadi *et al*.^36^ also compared the immediate effects of KT (*n* = 29) versus friction massage (*n* = 29) on latent UT muscle MTrPs by applying the space correction technique for 3 days. Contrary to other studies and to their own expectations, they showed a significant decrease in PPT values after treatment and suggested that perhaps KT and friction massage cause pain in latent MTrPs, which may become activated and evolve into active trigger points, thus decreasing the PPT. Furthermore, they also speculated that KT could have increased patient awareness of their pain in these potential trigger points, therefore contributing to lower PPTs.

In our study, despite reaching statistical significance in the post-treatment measurement in both groups (KT and sham) – presumably via the gate-control stimulation mechanism^23^ – KT had a small and clinically unmeaningful effect on PPT in patients with latent MTrPs. Indeed, this therapy did not reach the minimal clinically important difference of 4.5 N/cm^2^ reported for this variable for individuals with chronic neck pain^46^. Furthermore, this effect was not maintained after 72 h in these participants and was even lower (not reaching statistical significance) in those with active MTrPs. Accordingly, the absence of clinically relevant changes in PPT was not accompanied by improvements in the active ROM of the cervical spine in our study. These findings are consistent with several systematic reviews exploring how KT affects a variety of musculoskeletal pathologies^20–24,26^ which concluded, with a moderate degree certainty, that KT is just as clinically effective as sham tape in the short-term. The most important differences in our randomized sham-controlled trials were that we included more participants (trial A) and reached conclusions based both on statistical significance and the magnitude of the effects of the KT. Several trials have studied these effects in a variety of conditions, however it appears that the presentation of overestimated conclusions based only on statistical significance is not uncommon^26^.

Finally, we must highlight the fact that MTrPs may present in any muscle and the effects of KT can vary in different muscles; therefore, our findings should be interpreted with caution because we only considered the UT. In addition, the age range used in our final study participant population was between 18 and 32 years only, and so our results cannot be generalized to adults aged older than this threshold. Our work was also limited by the short-term nature of the treatments and because we did not collect any follow-up data or assess patient disability.

## Conclusion

Overall, our results do not provide any evidence for the usefulness of the space-correction KT technique in the treatment of patients with latent or active UT myofascial trigger points.
